# Supplementary material for: Clinical Validity of Anti-Proteinase 3 Antibodies in Patients with Inflammatory Bowel Disease: A Short Meta-Analysis
Source: Diagnostics (Basel). 2023 Dec 16;13(24):3682. doi: 10.3390/diagnostics13243682 (PMC10742424; doi:10.3390/diagnostics13243682)
Supplement: Supplementary file 1 [file diagnostics-13-03682-s001.zip › diagnostics-2727503-supplementary.pdf]

Supplementary Figure S1. Electronic searches.

PUBMED SEARCH	
**Search Name:**	anti-PR3 antibodies in IBD
**Date Run:**	June 6, 2023

ID	Search	Results	Hits
#1	Search: ((Inflammatory bowel disease[MeSH Major Topic]) OR (Crohn´s disease[MeSH Major Topic])) OR (Ulcerative colitis[MeSH Major Topic])	83912	*TARGET CONDITION*
#2	Search: (((PR3-ANCA[Title/Abstract]) OR (Anti-PR3 antibodies[Title/Abstract])) OR (Anti-proteinase 3 antibodies[Title/Abstract])) OR (cANCA/PR3 antibodies[Title/Abstract])	900	INDEX TEST
#3	Search: #1 AND #2	21	
	**Pubmed results**	**21**	

SCOPUS SEARCH	
**Search Name:**	anti-PR3 antibodies in IBD
**Date Run:**	June 6, 2023

ID	Search	Results	Hits
#1	inflammatory AND bowel AND disease OR crohn´s AND disease OR ulcerative AND colitis	171744	*TARGET CONDITION*
#2	anti-pr3 AND antibodies OR anca-pr3 OR anti-proteinase 3 antibodies OR canca/pr3 AND antibodies	662	INDEX TEST
#3	#1 AND #2	80	
	**Scopus results**	**80**	

WEB OF SCIENCE	
**Search Name:**	anti-PR3 antibodies in IBD
**Date Run:**	June 6, 2023

ID	Search	Results	Hits
#1	((AB=(Inflammatory bowel disease)) OR AB=(Crohn´s disease)) OR AB=(Ulcerative colitis)	82986	*TARGET CONDITION*
#2	(((AB=(anti-PR3 antibodies)) OR AB=(anti-proteinase 3 antibodies)) OR AB=(cANCA/PR3 antibodies)) OR AB=(PR3-ANCA)	916	INDEX TEST
#3	#1 AND #2	48	
	**Web of science results**	**48**	
